# Deep Learning Vision System for Quadruped Robot Gait Pattern Regulation

**DOI:** 10.3390/biomimetics8030289

**Published:** 2023-07-03

**Authors:** Christyan Cruz Ulloa, Lourdes Sánchez, Jaime Del Cerro, Antonio Barrientos

**Affiliations:** Centro de Automática y Robótica (CSIC-UPM), Universidad Politécnica de Madrid—Consejo Superior de Investigaciones Científicas, 28006 Madrid, Spain; christyan.cruz.ulloa@upm.es (C.C.U.); lourdes.sanchezl@alumnos.upm.es (L.S.); j.cerro@upm.es (J.D.C.)

**Keywords:** biologically inspired robotics, quadruped robots, convolutional neural networks, robotics vision, transfer learning

## Abstract

Robots with bio-inspired locomotion systems, such as quadruped robots, have recently attracted significant scientific interest, especially those designed to tackle missions in unstructured terrains, such as search-and-rescue robotics. On the other hand, artificial intelligence systems have allowed for the improvement and adaptation of the locomotion capabilities of these robots based on specific terrains, imitating the natural behavior of quadruped animals. The main contribution of this work is a method to adjust adaptive gait patterns to overcome unstructured terrains using the ARTU-R (A1 Rescue Task UPM Robot) quadruped robot based on a central pattern generator (CPG), and the automatic identification of terrain and characterization of its obstacles (number, size, position and superability analysis) through convolutional neural networks for pattern regulation. To develop this method, a study of dog gait patterns was carried out, with validation and adjustment through simulation on the robot model in ROS-Gazebo and subsequent transfer to the real robot. Outdoor tests were carried out to evaluate and validate the efficiency of the proposed method in terms of its percentage of success in overcoming stretches of unstructured terrains, as well as the kinematic and dynamic variables of the robot. The main results show that the proposed method has an efficiency of over 93% for terrain characterization (identification of terrain, segmentation and obstacle characterization) and over 91% success in overcoming unstructured terrains. This work was also compared against main developments in state-of-the-art and benchmark models.

## 1. Introduction

The research and development of bio-inspired quadruped robots have evolved in recent decades, resulting in robots with a great capacity for mimicry and locomotion modes inspired by animal behavior in nature. In these developments, intelligence systems should be highlighted. Biomimicry allows us to solve complex problems, such as moving through complex environments, a topic currently of interest at the research level [1].

Among the main challenges and limitations of this type of robot is its movement through unstructured terrain, with the presence of debris. In this way, terrestrial animals such as horses [2] and snakes [3] have inspired several robotic developments, allowing them to move with great agility in nature.

On the other hand, search-and-rescue robotics arise from the need to assist rescue brigades in interventions at post-disaster events, seeking to protect lives and help detect victims in the environment [4,5]. Historically, bio-inspired robots, such as those of the caterpillar type, have been used in this type of intervention, including: the United States (Twin Towers, 2001) [4,6], Japan (Fukushima, 2011) [7], Italy (Amatrice, 2016) [8] and Mexico (2017) [9].

The rise of quadruped robots has made it possible to explore new alternatives for exploration and displacement in rustic terrain, given their great agility, fast response times, omnidirectional movement, and ability to perform even in terrains where robots with conventional locomotion systems (wheels or caterpillars) are not able to [10].

Quadruped robots currently use LiDAR-based systems to locate and identify the terrain [11], which faces disadvantages in accurately characterizing stable zones and surmountable obstacles, or they generalize the terrain using specific contact sensors for the characterization of materials [12,13]. However, real scenarios, by nature, are constantly changing, unstructured and unstable, which represents a challenge within state-of-the-art robotic systems in adjusting gait patterns. This will be addressed in the first approach of this study. On the other hand, there have been methods developed to generically identify the type of terrain [14,15,16], but the problem of characterizing its elements is not focused, and the obstacles that represent a challenge for mobility on these surfaces are not structured.

The main contribution of this work is overcoming the challenges of unstructured terrain using the ARTU-R quadruped robot (A1 Rescue Task UPM Robot), automatically adjusting the kinematic and dynamic parameters of its gait patterns based on the identification of the terrain using a central pattern generator and characterization of the terrain obstacles using neural networks for gait pattern regulation.

To this end, a simulation phase was started in environments by ROS-GAZEBO to validate a virtual model of the robot, using the gait patterns studied in dogs to determine the relevant parameters of the walk, which would later be adjusted based on the information output from the neural network. The adjustments of the kinematic and dynamic parameters of the robot’s gait patterns were made based on the automatic analysis of the terrain type (gravel, earth or grass) and the type of obstacles in it.

This automatic recognition and semantic segmentation of the environment was carried out by training a convolutional neural network (YOLOv8) using a dataset of more than 1700 images. Tests were carried out in real environments to validate the proposed method, with successful results in overcoming unstructured terrain with the robot.

This paper is structured as follows: Section 2 shows the most relevant works on terrain identification and characterization. Section 3 details the materials and methods used. Section 4 describes the experiments and results. Finally, the main findings are presented in Section 5.

## 2. Related Work

### 2.1. Automatic Terrain Identification Robotic Systems

The identification and characterization of environments is a widely studied problem in robotics perception to determine the specific characteristics of the environment in order to define advance and displacement strategies. The main methods used for this task are LiDAR-type sensors, RGB-D cameras and sensors for material characterization [17]. In this way, two subsections can be established for identifying terrain: sensors that require contact with the soil material and visual-type sensors.

#### 2.1.1. Identification Based on Contact Sensors

Common terrain identification methods use specific sensors built into the robot’s legs. These sensors are distributed to identify different types of materials [12,13,18,19] and allow locomotion parameters to be adjusted for the robots depending on the terrain. For their part, other robots with hexapod legs use force/torque sensors and Bayesian-type classifiers to determine terrain type [20].

Others base their functionality on vibration systems combined with linear discriminant analysis to characterize the terrain, mainly types similar to the Martian rover [21,22,23].

Contact-based systems for land identification show promising results when there is complete contact, but they face a series of problems when there is no complete contact due to debris or there is a generalization of the entire terrain based on local measurements. Another disadvantage is that it requires stable information.

#### 2.1.2. Identification Based on Visual Perception

Most works related to RGB-D type sensors [24,25] are limited to extracting characteristics and identifying objects or planes [26,27,28].

On the other hand, lidar-type sensors are used for re-constructing 3D environments and the semantic identification of areas and objects based on geometries and the extraction of planes and surfaces [29,30,31]. However, most developments are limited to the extraction of plans [32,33].

Some works use RGB imaging and neural networks for terrain identification, such as [14,34,35,36,37,38]. However, there is a lack of systems for detecting and characterizing obstacles or surmountable zones for the robot, which is a fundamental factor for defining walking modes and areas to avoid.

Although the methods based on visual systems are robust and reliable in characterizing the environment, they show some disadvantages. Thus, lidar-based systems cannot infer or provide information about the rigidity or stability of the ground or obstacles.

In this sense, the proposed method seeks to implement a proof of concept by using neural networks trained with a starting criterion of surmountable or non-surmountable obstacles, considering size and location, given by a user.

This first phase consisting of the detection and characterization of terrain allows the robot intelligence systems to develop preliminary strategies to address unstructured environments, by adjusting the modes of locomotion as progress is made, according to the structure of the environment.

### 2.2. Gait Pattern Adjustment of Bioinspired Quadruped Robots

Biomimetic intelligence systems allow solving complex problems such as moving across complex environments, which is currently of interest at the research level [1].

Some works combine several contact-type sensors to define the displacement of quadruped robots based on the optimization of forces and torque control strategies [39,40,41,42]. On the other hand, some developments integrate vision systems to achieve first attempts at traversing terrain with a quadruped robot by using terrain mapping tools in controlled environments [43].

The work “A Review of Quadruped Robots and environment perception” highlights one of the main problems to be addressed within this area, which is the identification of the terrain, which must be interpreted in a bioinspired way to be addressed satisfactorily [44].

There are also other works related to the regulation of gait parameters for robots with legs, such as in ref. [45], which establishes that moving across unstructured terrains with a single gait pattern is complex. This work proposes a system that regulates the gait patterns of a hexapod robot and includes a method based on a fixed gait pattern and an adjustable one based on the inclination of the terrain. In work developed by Zenfer, it is proposed to adjust the gait patterns of a hexapod robot based on the terrain identified by a monocular camera [46].

An analysis of the kinematics of a leg of a quadruped robot is presented in [47]. In [40], one of the first developments in gait pattern regulation is shown, focusing on trot and gallop by using contact sensors on the robot’s legs as feedback.

On the other hand, a method that feeds back the gait patterns of a spider-type robot based on the terrain detected with an RGB-D vision system is proposed in [15]. At the same time, Gong proposes a method for extracting the gait patterns of quadruped animals based on their pose [48]. Chen proposes a method for pattern matching a robot with legs–wheels [49]. A method to adjust the patterns in quadruped robots according to the touchdown times of swing feet is evaluated in [50].

Several relevant works stand out within the state of the art. However, the method proposed by the authors, which combines neural networks to identify the terrain from an RGB image so as to define which obstacles/zones are surmountable, has not been addressed so far to adjust the gait patterns.

## 3. Methodology

### 3.1. Materials

**Figure 1:**
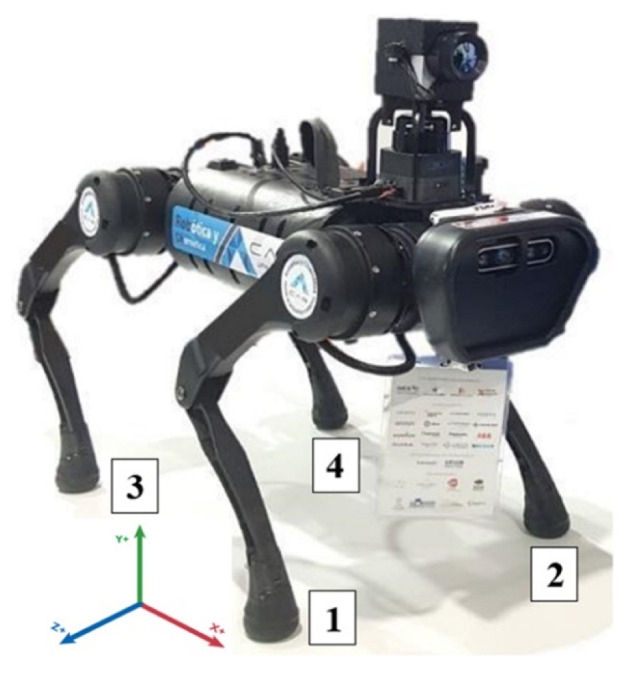
ARTU-R quadruped robot (A1 Rescue Task UPM Robot), equipped with sensory equipment for hostile environments. Numbers on the legs are assigned for identification throughout the manuscript. Source: authors.

The main equipment used for this work is ARTU-R, a quadruped robot, shown in Figure 1. Its sensory system comprises the elements described in Table 1. This robot relies on 12 brushless motors distributed among its four legs to moves. The main characteristics of these motors are a weight of 0.605 Kg, a maximum torque of 33.4 N.m and an Encoder of 15 bits used to determine the position of each link.

A Gazebo simulation environment executed in a previous phase on a high-powered computer (MSI-10th Gen, GTX-1660Ti) allows the simulation of different parameters and configurations of movement.

### 3.2. Kinematic Modeling of the Legs

The quadruped robot used in this work has three degrees of freedom per leg, which amounts to a total of twelve degrees of freedom. Each limb is made up of three links. The problem will be subdivided into two sections to find the inverse kinematics of the system.

**Figure 2:**
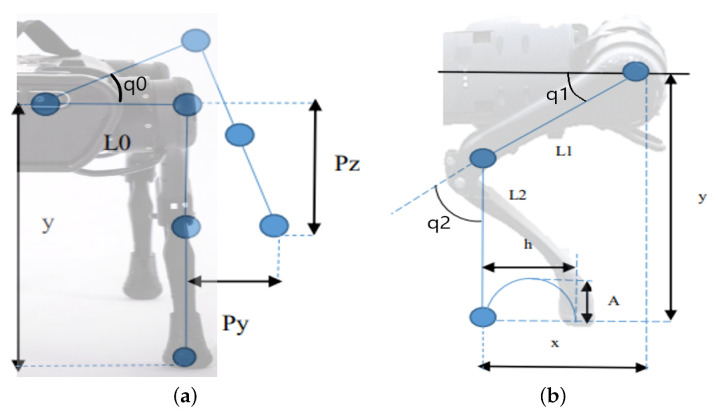
Views and parameters of the kinematic model of the robot. Source: authors. (**a**) Front view of the kinematic model; (**b**) lateral view of the kinematic model.

In the first step, the situation of one of the legs in the frontal plane (YZ) in Figure 2a will be analyzed to find the angle q0 as a function of the distances Pz and Py and the length L0, using Equation (1).

In the second part of the kinematics calculation (Figure 2b), the triangle formed on the limb’s lateral side is considered. Thus, it will be possible to obtain the angles q1 and q2 from the x and y values. Equations (2) and (3) show the relationship for calculating these angles.

These expressions are found as a function of the parameters of the forward step of the robot given by (h, A), shown in Figure 2b, that will be combined with the outputs of the neural network to generate the adaptive movement.

(1) $$q_{0}=tan^{-1}\left(\frac{y_{f(A)}}{L_{0}}\right)-tan^{-1}\left(\frac{P_{z}}{P_{y}+L_{0}}\right)$$

(2) $$q_{2}=cos^{-1}\left(\frac{{\left(x_{f(A)}\right)}^{2}+{\left(y_{f(A)}\right)}^{2}-L_{1}^{2}-L_{2}^{2}}{2\cdot L_{1}\cdot L_{2}}\right)$$

(3) $$q_{1}=tan^{-1}\left(\frac{y_{f(A)}}{y_{f(h))}}\right)-tan^{-1}\left(\frac{L_{2}\cdot sen\left(q_{2}\right)}{L_{1}-L_{2}\cdot cos\left(q_{2}\right)}\right)$$

#### Iterative Configuration of Gait Patterns

**Figure 3:**
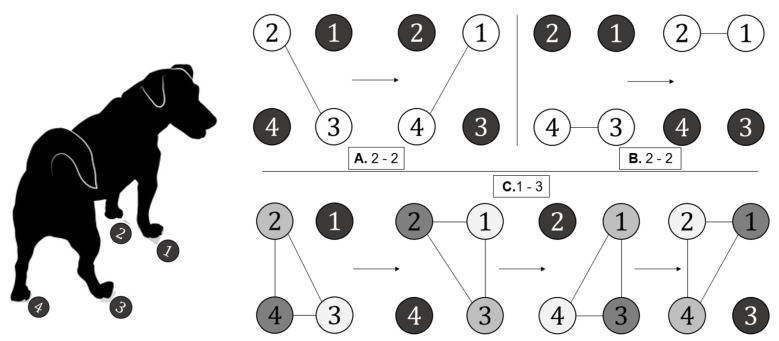
Synthesis of the gait patterns studied. A: 2-2 Altern, B: 2-2 Gallop, and C: 1-3. Source: authors.

Once the leg model is obtained, it is adapted to the different base gait patterns analyzed in dogs (Figure 3), where each paw is identified from 1 to 4 according to Figure 1. There are three types of patterns: configuration A, 2-2 alternate, where 1 + 4 and 2 + 3 are moved synchronously; configuration B, 2-2 gallops alternative (movement of 1 + 2 and 3 + 4); and configuration C, 1-3, that has four phases, leaving a support polygon of three legs while the other one is in the air. It is worth noting that the three paws on the ground must continue in a phased synchronous movement to generate an advance. This four-time-phase advance for each leg is illustrated in grayscale for better visualization in Figure 3.

The 2-2 gait pattern has great importance. This type of movement has different variants depending on how the two pairs of limbs are organized. On the one hand, there is the alternate mode, in which one front and one hind leg advance simultaneously. In this method, two legs move, leaving the other two static, and later, at the end of the journey of the first one, the other two start-up.

The trajectories to be followed by the hoof have been proposed as a positive sinusoidal curve, with amplitude (A) and half the period equal to the step (h); the parameters shown in Figure 2b.

### 3.3. Test Environments and Parameters

The simulations carried out in this work have been applied to different types of terrain, all encompassed in the so-called orange sand, according to the Institute of Standards and Technology (NIST). Taking NIST as the regulatory entity, the proposed environments are classified into three types of arenas, yellow, orange and red, each one with a different level of complexity [51]. Accordingly, the scenarios in this work contain moderate obstacles, slight slopes and different consistencies of soils.

#### 3.3.1. Simulated and Real Environments

The simulation phase allows the analysis of the defined gait patterns to evaluate their functionality in unstructured terrain. The ROS-GAZEBO simulator is used, which recreates both the physical and dynamic conditions of the environment. It also allows the integration of the CAD model of the robot with Ros-Control packages.

**Figure 4:**
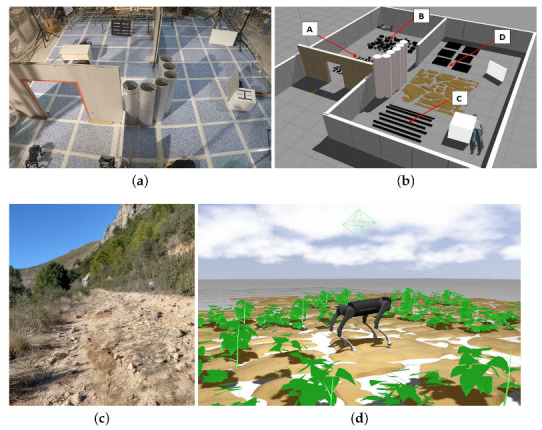
Real and simulated test environments. Source: authors. (**a**) Real scenario (CAR robotics arena) with different instances for testing; (**b**) indoor simulated scenario in Gazebo for test execution; (**c**) outdoor real scenario; (**d**) outdoor simulated scenario with the robot model.

The simulation in Gazebo is reconstructed based on the CAR-Arena of the UPM to have parity in the concordance of environments. Both environments are shown in Figure 4a,b, respectively. In the same way, outdoor environments are reconstructed for this testing phase according to those used later, shown in Figure 4c,d.

The indoor environment (Figure 4a,b) consists of different facilities with four types of floors. The first (A), located when crossing the door, has small stone-type rubble that does not exceed 1–3 cm. The second (B), located in the rear-left area, relies on larger rubble pieces (5–9) cm. In the next room, an area (C) with branch-type rubble and an area with unevenness (D) are mainly distinguished.

#### 3.3.2. Type of Tests

A series of tests were carried out in each different type of terrain, analyzing the time required to complete each one of the routes, the stability that the robot shows while facing different obstacles and the maximum distance.

The best gait patterns for each type of scenario and their most appropriate parameters were extracted, and predefined as functions for their extrapolation to the real scenario testing phase.

Tests in real environments are started by identifying the environment and characterizing the debris in terms of size, relative position and distance. Based on these data, the adaptive algorithm for the compensation of the trajectory of the movement of the leg is adjusted for the advance through the terrain. Different kinematic parameters have been analyzed to evaluate the success of each test.

### 3.4. Convolutional Neural Networks for Identification and Characterization of the Environment

The YOLOv8 convolutional neural network was used to develop this work since it shows several advantages over its predecessors in addition to classification and detection. The main innovative element in this version of CNN is the segmentation layer on the detected objects that it incorporates, which allows extracting more precise information, mainly the centroid, based on the distribution of the specific area of the object.

#### 3.4.1. Datasets and Network Training

The training of the neural network starts with generating a dataset of images captured in outdoor terrain (grass, gravel, and dirt road) with different conditions and various obstacles. The dataset comprises more than 1700 images in the authors’ Github repository (Appendix A).

The labeling phase was carried out using the following labels to identify the terrain and the variety of obstacles existing in it: gravel, dirt road, grass, obstacle—surmountable and obstacle—not surmountable.

The dataset was divided into three groups according to the following percentages: training (82%), validation (12%) and testing (6%).

The training phase was carried out on a high-performance computer in the Anaconda environment. The number of epochs required for training was 145. Once the model was obtained, its effectiveness was evaluated in terms of precision, recall and accuracy according to Equations (4)–(6). The components of the true positive (TP), false positive (FP), false negative (FN) and false positive (FP) equations are derived from the inferences in the network detection. These metrics enable the authors to establish curves and analyze elements of the effectiveness of the network, such as the confusion matrix.

(4) $$\mathrm{Precision}=\frac{TP}{TP+FP}$$

(5) $$\mathrm{Recall}=\frac{TP}{TP+FN}$$

(6) $$\mathrm{Accuracy}=\frac{TP+TN}{TP+FP+TN+FN}$$

#### 3.4.2. Automatic Adjustment of Patterns Based on the Neural Network Processing

**Figure 5:**
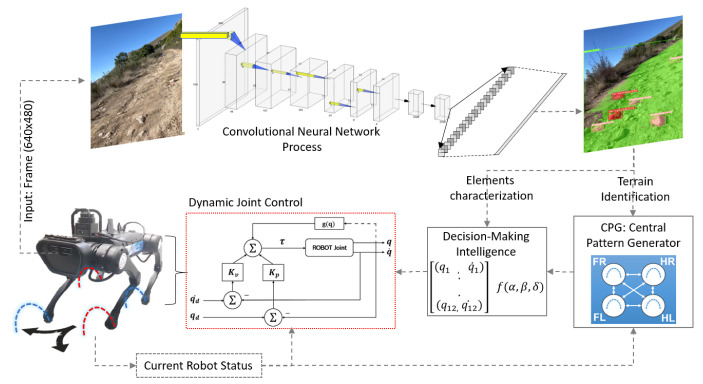
Schematic diagram of the implemented system. Source: authors.

Figure 5 shows in detail each subsystem of the implementation developed for this work. After the simulation phase, some basic gait patterns able to work in irregular terrain are defined that can work for irregular terrain. The next stage corresponds to adjusting these patterns based on the changing environment.

The cyclical process begins with the image captured by the robot. It is processed with the trained model and characteristics of the environment (terrain ID and obstacle characterization) are extracted, which feed the pattern-matching system. The new adjusted values are sent to the predefined dynamic controller of the robot. The computational frequency required for processing all subsystems is 10 Hz.

The parameters provided by the network are, on the one hand, the type of terrain (used to define the basic walking pattern in the Central Pattern Generator—CPG) and, on the other hand, the obstacle list (Obst). The centroid (relative to the central position of the camera), radio (Radio) and distance (dist) are obtained concerning the frame where the robot camera is allocated—in this case, in front of the robot.

Since the number of objects detected could be different for each iteration, and they could be false detections or wrong estimates for the type of terrain, incremental dynamic matrices are used to generate greater confidence in the updating values obtained.

These parameters are used in Equations (7)–(11) to adjust the gait patterns by varying the amplitude and/or its step length according to the dynamic stiffness of the joints. Equations (7) and (9) provide the new values of the marching pattern ($A_{temp},h_{temp}$), and Equations (8) and (10) adjust these values in a complementary way to the base pattern due to the identified terrain, together with Equations (1)–(3). Finally, Equation (11) is used to define the stiffness of joint articulations. Algorithm 1 details the functional structure of the process used for the proposed method.

(7) $$\left(A_{temp},k_{p1}\right)=\frac{\sum_{i=1}^{n}\left[Obst_{centroid\to x}+Radio_{Obst\to Major}\right]*dist_{Obst}}{n}$$

(8) $$f\left(A\right)=A_{def}+\left(\bar{A_{temp_{def}}}\right)$$

(9) $$\left(h_{temp},k_{p2}\right)=\frac{\sum_{i=1}^{n}\left[Obst_{centroid\to x}+Radio_{Obst\to Minor}\right]*dist_{Obst}}{n}$$

(10) $$f\left(h\right)=h_{def}+\left(\bar{h_{temp_{def}}}\right)$$

(11) $$K_{p}=k_{p1}+k_{p2}$$

**Algorithm 1** Quadruped Robots Gait Pattern Regulation
1:

Data:

2:$im_{RGB}\leftarrow$ RGB image [640x480]3:

$Robot_{Joints-pose}\leftarrow$ $q_{\left[1-12\right]}$

4:

$Robot_{Joints-vel}\leftarrow$ ${\dot{q}}_{\left[1-12\right]}$

5:

$Robot_{pose(xyz)-orient(rpy)}\leftarrow$ $IMU_{estimation}$

6:

Result:

7:

$\left[q_{d_{\left[1-12\right]}},{\dot{q}}_{d_{\left[1-12\right]}},\tau_{\left[1-12\right]}\right]$

8:**function**Terrain_Processing(im)         ▹ CNN vision-based terrain processing9:    $CNN_{based-algorithm}\leftarrow im$10:    return $\left[Terrain_{ID},Obstacles_{class,size,pose}\right]$11:
**end function**
12:**function**Gait_Pattern(TerrainDetected)         ▹ Gait base pattern generator13:    $\left[q_{d_{\left[1-12\right]}},{\dot{q}}_{d_{\left[1-12\right]}},\tau_{\left[1-12\right]}\right]\leftarrow IK\_Solver_{terrain/exp-based}$14:    return $\left[GaitPattern_{\left[q,\dot{q},\tau \right]}\right]$15:
**end function**
16:**while**$im_{RGB}$ and start**do**            ▹ Main Loop17:    Robot←stand_position18:    eval (TERRAIN_PROCESSING←$im_{\left[RGB\right]})$19:    **if** Terrain−ID not null **then**20:        eval (GAIT_PATTERN←Terrain−ID)21:        **if** Obstacles in terrain not null **then**22:           eval_adjusted_pattern $\left(\left[f\left(A\right),f\left(h\right),K_{p}\right]\leftarrow Obstacles_{\left[number,pose,size\right]}\right)$23:           update_control_variables $\left(\left[q_{d_{\left[1-12\right]}},{\dot{q}}_{d_{\left[1-12\right]}},\tau_{\left[1-12\right]}\right]\leftarrow \left[f\left(A\right),f\left(h\right),K_{p}\right]\right)$24:           $Robot_{Controller}\leftarrow \left[q_{d_{\left[1-12\right]}},{\dot{q}}_{d_{\left[1-12\right]}},\tau_{\left[1-12\right]}\right]$25:        **else**26:           $Robot_{Controller}\leftarrow \left[q_{d_{\left[1-12\right]}},{\dot{q}}_{d_{\left[1-12\right]}},\tau_{\left[1-12\right]}\right]$27:        **end if**28:    **else**29:        Robot←stand_position30:    **end if**31:
**end while**



A PD (proportional-derivative) controller with gravity compensation is used for each joint. One of the most responsive parameters is the constant Kp or stiffness constant since the behavior of the entire leg in front of an obstacle depends on the magnitude. Thus, if high Kp values are set, a small margin to generate adaptability in the face of the obstacle is obtained. Due to this, Kp values are specified for each joint at every step in order to obtain adequate adaptability. These values are directly proportional to the number of obstacles and environment.

## 4. Results

### 4.1. Simulation Analysis

**Figure 6:**
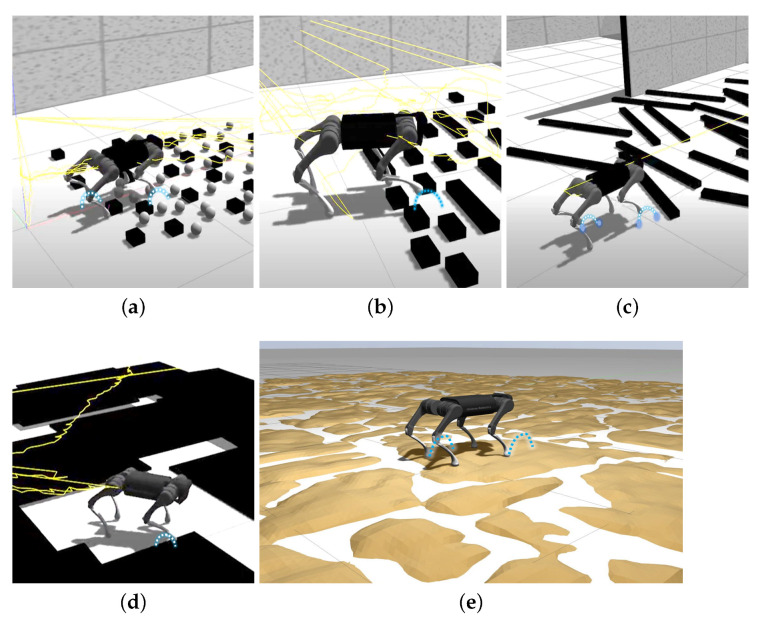
Tests executed in simulation. The trajectory described by the end of the robot’s leg is shown in blue. Source: authors. (**a**) Conf: 2-2 Debris: small. (**b**) Conf: 1-3 Debris: medium. (**c**) Conf: 2-2 Debris: branches. (**d**) Conf: 1-3 Unevenness. (**e**) Conf: 1-3 Unevenness.

In the first part of validating the implemented method, the three types of gait pattern are evaluated on the different scars (Simulator Common Architecture Requirements Standards) in simulation to define the best initial set-up as the basis for transfer learning. Figure 6 shows the results of the simulations, where the robot model can be seen moving in the different scenarios. The hoof trajectory corresponding to each gait pattern is shown in blue.

Figure 6a shows the Gazebo simulation on terrain with small prismatic and spherical obstacles, corresponding to the type of soil (A) detailed in Section 3.3.2. It is found that the most favorable gait pattern for this soil is 2-2. The average time for the robot to reach the goal in this scenario is 14.2 s.

Figure 6b–e show the rest of the tests carried out. Table 2 summarizes the results of the tests carried out in terms of time, percentage progress and distance covered.

The most appropriate gait mode is the so-called alternate 2-2. When following a 1-3 pattern, the robot is able to advance but cannot fully overcome the obstacles. Moreover, the 2-2 gallop mode turned out to not be the most suitable for this type of scenario.

Accordingly, the alternate 2-2 mode is used as a base pattern for the tests carried out with the real robot; the adjustment of this pattern will be simultaneously executed based on the perceptible environment of the robot.

### 4.2. Evaluation of Detection and Autonomous Characterization of Real Terrain

#### 4.2.1. Analysis of the Convolutional Neural Network Efficiency

**Figure 7:**
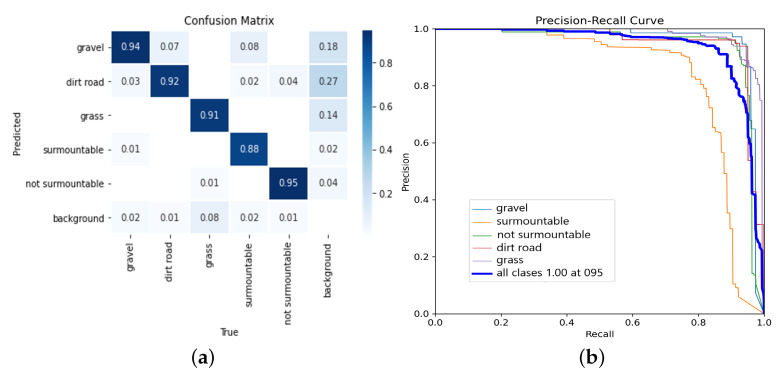
Evaluation of the trained neural network model. Source: authors. (**a**) Confusion matrix for the trained model. (**b**) Precision–recall curve for the trained model.

Figure 7a shows the confusion matrix for the trained model of the neural network. The main diagonal shows high values close to one, indicating a high confidence level for detecting each class.

The best identification rate was obtained for the terrain with gravel. In the same way, the obstacles that cannot be overcome are well identified (95&), a significant factor that acknowledges that due to the geometric restrictions of the robot or the arrangement of the obstacle in the environment, it cannot be overcome, and reactive movements are generated to avoid it.

On the other hand, Figure 7b shows the precision–recall curve, which shows the trend of stability in detection precision and its subsequent decline. The values obtained for all the classes are uniform and over 90 %, except for the class of surmountable obstacles, which provides a value of 88%.

#### 4.2.2. Evaluation of the Environment Characterization

**Figure 8:**
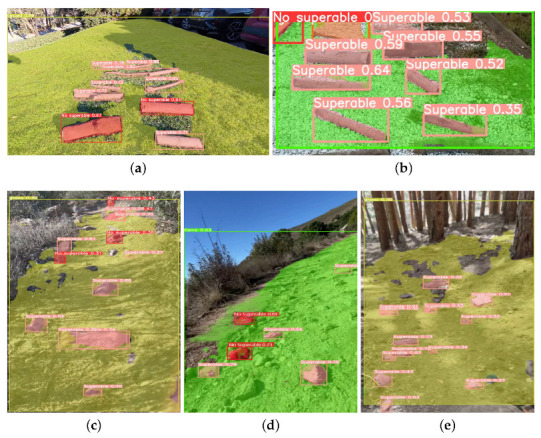
Analysis of the terrain characterization through the trained neural network. Source: authors. (**a**) Outdoor scenario analysis—grass with obstacles. (**b**) Outdoor scenario analysis—gravel with obstacles. (**c**) Outdoor scenario analysis—dirt road with obstacles. (**d**) Outdoor scenario analysis—gravel with obstacles. (**e**) Outdoor scenario analysis—dirt road obstacles.

The evaluation results for the outdoor scenarios are illustrated in Figure 8. This figure shows the different overlapping layers on the analyzed image, the bounding boxes, the classes and the precision percentages for each detected obstacle.

Figure 8a shows a first environment with grassy soil, segmented in green with different obstacles. Those that can be overcome are shown in pink, and those that cannot be overcome due to their size or instability in red. The areas detected for both environment and obstacles have a high efficiency. This is mainly because the environment is quite structured, similar to the one in Figure 8b (gravel).

On the other hand, Figure 8c–e correspond to terrains with rough conditions, where both terrain and obstacles are marked as layers of colors. In these cases, the percentage of success in detection and characterization also obtains a high confidence index in the implemented method.

#### 4.2.3. Analysis of the Vision Method Regarding the State of the Art

The most important benchmarks that contain images of outdoor environments and semantic segmentation of the terrain are MSeg [52], TAS-NIR [53], TAS500 [54], TimberSeg [55] and RELLIS-3D [56].

Most of these benchmarks directly catalog the terrain as “roads”, “sidewalks” or “vegetation” but do not go into detail about the type of terrain or what it is, or more specific characteristics that it may contain, such as obstacles. The rest of the tags generalize urban environments in a certain way, such as traffic light, traffic sign, vegetation, terrain, sky, person, rider, car, etc.

For the development of this approach, different external scenarios corresponding to the [54,55] benchmarks are evaluated using the introduced neural network model to verify its effectiveness. The main results obtained are related to both the terrain detected and the presence of obstacles, as well as the percentage of terrain segmentation concerning the benchmark.

**Figure 9:**
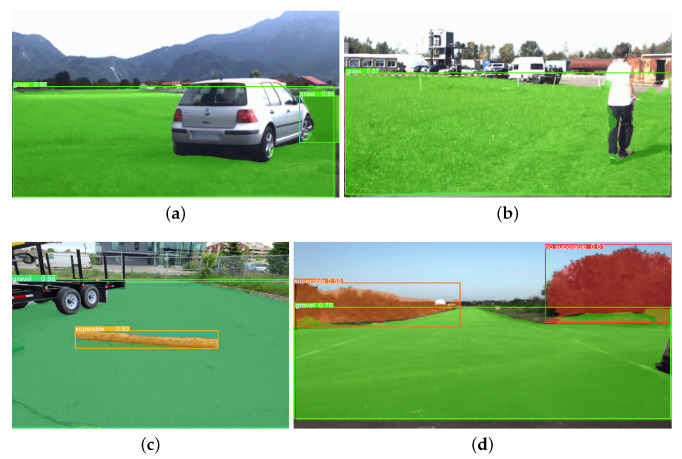
Evaluation of the trained neural network model over datasets [54,55] for terrain and obstacle detection. Source: authors. (**a**) Evaluation of terrain with the proposed detection model on the dataset [54]. (**b**) Evaluation of terrain with the proposed detection model on the dataset [54]. (**c**) Evaluation of terrain and obstacles with the proposed detection model on the dataset [55]. (**d**) Evaluation of terrain and obstacles with the proposed detection model on the dataset [55].

Figure 9 shows the result of detecting terrain type and semantic segmentation. However, elements such as the sky, people or cars are not detected. It should be noted that the model is not focused on that type of element, only on the ground and possible obstacles. Figure 9a,b shows the correct identification of the soil (grass) with an average precision of 0.88% and 0.94% of the total segmented area concerning the original benchmark.

On the other hand, Figure 9c,d shows the recognition of the soil, identified as gravel, and in the first case, an obstacle identified as surmountable. In the second case, the bushes are identified as obstacles. However, these are outside the identified terrain, so they are not part of the displacement environment. In both cases, the average percentage of detection of the segmented space concerning the benchmark is 93%.

On the other hand, a comparison is established, based on different metrics of the proposed method against those existing in the state-of-the-art vision system for land characterization. This approach is shown in detail in Table 3 and shows the strengths of the proposed method.

As the first result of this comparison, it can be established that most of the previously developed works were focused on the characterization of the terrain, either with a subsequent step of semantic segmentation or not. Moreover, there is a lack of systems for characterizing components such as debris, information that is valuable for decision making in the field of outdoor robotics.

Other conventional methods for environment identification based on point clouds generally use traversability maps [58,59]. However, this method lacks relevant environmental information, such as the stability of areas and obstacles. Identifying terrains using the proposed method provides a better perception of the environment and its stability. Using traversability maps, the environment is considered compact, and pass/no-pass zones are established to generate planning routes based on different heights or slopes.

### 4.3. Analysis of Results Working with the Real Robot

**Figure 10:**
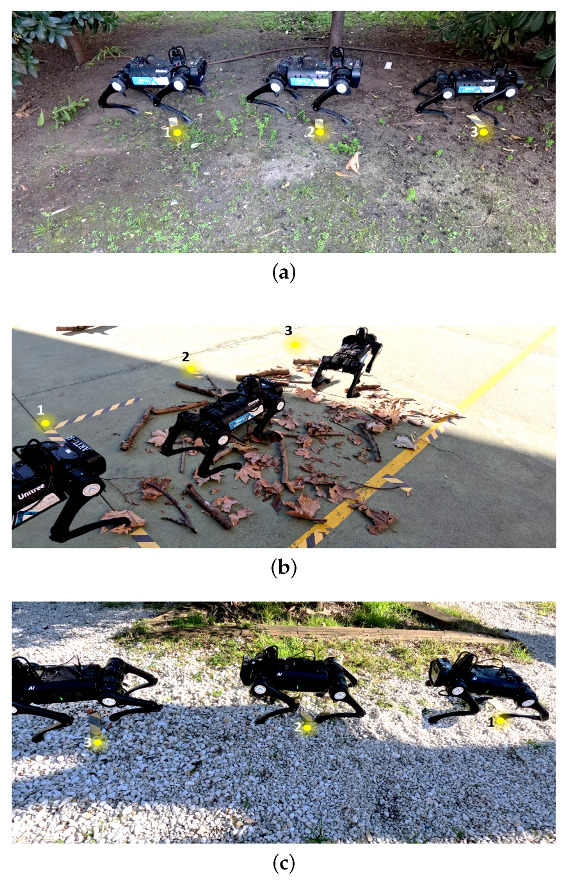
Evaluation of the robot’s performance in overcoming different types of terrain. Source: authors. (**a**) Overcoming of external terrain, type dirt road. (**b**) Overcoming of external terrain with obstacles. (**c**) Overcoming of external terrain, type gravel.

Tests were carried out mainly in outdoor environments to validate the joint operation of the proposed method. Figure 10 shows three scenarios where the tests were carried out. For the quantitative evaluation, an advance of two meters is considered. The initial points are marked with the number one, the intermediate steps as two and the final point as three.

Figure 10a corresponds to a dirt-road-type terrain. Figure 10b shows a scenario with several obstacles to overcome, while Figure 10a corresponds to a terrain with gravel. In the three scenarios, different superimposed frames of the robot moving along the path are shown.

Table 4 and Table 5 show the results corresponding to a series of tests carried out on different terrains, both with and without obstacles, respectively. It can be seen (as preliminary conclusions from these results) that the influence of terrain with obstacles increases the time required to complete the mission since the speed of the robot decreases. On the other hand, the pattern adjustment algorithm increases the mean values of the gait pattern to overcome these detected obstacles. Although the percentage of success decreases, the average pass rate for these areas is over 90.5%. This demonstrates the proposed method’s effectiveness in addressing unstructured terrain in this first approximation.

#### Comparison of Gait Pattern Adjustment Methods in the State of the Art

The developed comparison is shown in Table 6. The previously developed works focused on adjusting the gait patterns of different types of robots, not only quadrupedal ones. Those referring to quadruped robots mostly use the adjustment of patterns using contact sensors with the floor to evaluate the stability in an all/nothing way. Some other works already integrate RGB-D image processing to characterize the spatial depth in each step to adjust the march.

On the other hand, several works based on hexapod and spider robots are able to regulate their gait patterns (mostly alternate tripod) depending on the type of terrain using RGB sensors. However, a development similar to the proposed by the authors for regulating gait patterns based on visual processing and characterizing the environment has not been found in a specific way.

### 4.4. Joint Behavior

**Figure 11:**
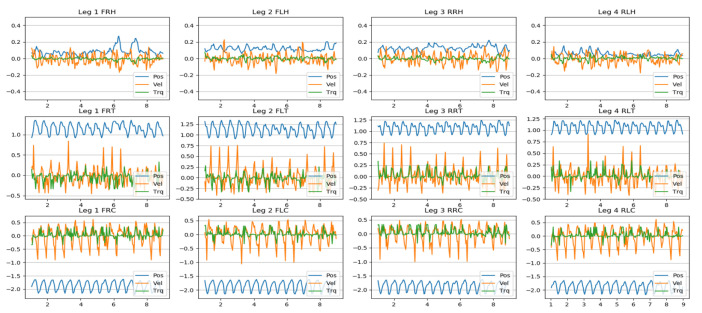
Joint behavior compared to the 2-2 configuration gait pattern with high amplitude (14 cm) and medium footprint (5 cm), for a dirt-road-type environment with obstacles for time t = 10 s.

Figure 11 shows the results of the joint behavior (angular position, velocity and torque) during the initial 10 seconds of displacement across the terrain test with obstacles in Figure 10. The units and nomenclature are described as position (Pos) (rad) (blue), velocity (Vel) (rad/s) (orange − scale * 0.1) and torque (Trq) (N.m) (green − scale * 0.01) of the joints.

The graphs show the behavior of each of the three joints, corresponding to the four limbs, according to the nomenclature in Table 7.

The oscillatory movement of the position can be highlighted especially in the thigh and calf joints, which generate progress according to the gait pattern. In contrast, the joint behavior of the hip position is more uniform. In the same way, the velocity graphs have similar oscillatory behavior in the same type of position.

There are notable variations, especially in the 6–8 s for the front legs. These variations are due to the obstacles in the environment, which prevent reaching the movement’s referential positions. The hip joint’s movement stands out, which acts in a reactive manner to adapt to the variability of the terrain.

## 5. Conclusions

In this article, a method is presented and validated to overcome unstructured terrain using a quadruped robot. This method uses robot-modeled gait patterns and automatic identification–characterization of the environment to adjust these gait patterns automatically.

The study of bio-inspired locomotion systems in quadruped animals has allowed their imitation by real robots. These movements and gait patterns have been combined with intelligent systems to adjust movement in unstructured environments. This knowledge is used for the initial training of neural networks, which has allowed carrying out successful displacement in unstructured terrain with the robot.

The simulation phase has allowed validating the imitation of the gait patterns of dogs and analyzing their effectiveness in different types of terrain with obstacles. In this way, the 2-2 alternate type of gait pattern is revealed as the most adequate to overcome environments with debris. This pattern is considered to feed into the central gait pattern generator, which serves as the basis for forward movements.

The autonomous visual identification of the terrain and the characterization of the obstacles using convolutional neural networks has shown high efficiency, with a high percentage of precision (>90%) in the location of obstacles in real dynamic environments. This method is compared with similar state-of-the-art and relevant benchmarks, obtaining optimal functionality results.

The proposed method offers an enhanced understanding of the environment and its stability for terrain identification by utilizing RGB images. In contrast, conventional approaches relying on point clouds often require traversability maps. Nevertheless, these maps suffer from a lack of environmental details, including area stability and obstacle information.

The proposed method based on vision has shown operation efficiency outdoors. It could be extrapolated to other robotic systems and autonomous navigation vehicles. Future lines of research and subsequent developments based on sensory fusion with lidar systems to obtain more precise measurements of the characterized environment could be delivered from it.

## Figures and Tables

**Table 1:** Materials for the proposed system implementation.

Component	Description
Unitree A1	Quadruped Robot
Nvidia Jetson Xavier-Nx	On-board Embedded System
Real-Sense	RGB-Depth Sensor
MSI1660-Ti Laptop	Computer for Simulations

**Table 2:** Gazebo simulation results.

Simulation Results					
**Scenario:**		**A.**	**B.**	**C.**	**D.**
**Gait Patterns**	**Repetitions:**	**30**	**30**	**30**	**30**
1-3	av. time (s)	18.2	21.1	17.7	48.3
	% advance	98.3	90.2	66.3	75.3
	av. distance (m)	3.4	1.9	2.9	3.9
2-2 alternate	av. time (s)	14.2	10.2	16.3	17.4
	% advance	98.4	98.1	73.2	81.5
	av. distance (m)	3.3	2.1	2.41	4.41
2-2 gallop	av. time (s)	25.1	9.2	14.6	13.6
	% advance	81.4	54.4	64.1	75.5
	av. distance (m)	2.3	2.1	2.41	3.41

**Table 3:** Comparison of the proposed method for terrain identification characterization concerning state-of-the-art methods. Meets: ✓. Fails: X.

Work	Terrain ID	Obstacle ID	Benchmark Test	Sensor	Tested on Robots	Semantic Segmentation
[35]	✓	X	X	RGB	X	X
[14]	✓	X	✓	RGB-D	✓	X
[34]	✓	X	✓	RGB	X	✓
[36]	✓	X	X	RGB	X	✓
[15]	✓	X	X	RGB-D	✓	X
[46]	✓	X	✓	RGB	✓	X
[16]	✓	X	X	RGB	X	X
[37]	✓	X	X	RGB-D	✓	✓
[57]	✓	X	X	RGB	✓	✓
[25]	X	✓	X	stereo camera	✓	X
[38]	✓	X	X	RGB	X	✓
Authors	✓	✓	✓	RGB-D	✓	✓

**Table 4:** Test results for a two-meter course on terrain with obstacles.

Scenery	Dirt Road	Compact Soil	Gravel	Grass
Number of Tests	15	12	12	12
Mean speed	0.09 m/s	0.11 m/s	0.11 m/s	0.12 m/s
Mean time	20.1 s	17.1 s	17.7 s	16.3 s
Average body height	23.1 cm	23.2 cm	22.1 cm	22.9 cm
Average step height (A)	14.7 cm	13.2 cm	14.7 cm	12.6 cm
Average step length (h)	6.5 cm	7.0 cm	7.1 cm	6.8 cm
Completion success rate	89%	91%	89%	93%

**Table 5:** Test results for a two-meter run on unstructured terrain.

Scenery	Dirt Road	Compact Soil	Gravel	Grass
Number of tests	15	15	15	10
Mean speed	0.19 m/s	0.23 m/s	0.2 m/s	0.19 m/s
Mean time	10.2 s	8.5 s	9.7 s	10.2 s
Average body height	25.6 cm	25.3 cm	23.1 cm	24.4 cm
Average step height (A)	10.7 cm	8.4 cm	10.7 cm	9.3 cm
Average step length (h)	7.3 cm	8.1 cm	9.1 cm	7.9 cm
Completion success rate	94%	100%	93%	100%

**Table 6:** Comparison of gait pattern adjustment methods. Meets: ✓. Fails: X.

Work	Robot	Visual Terr. Det.	Visual Obst Det.	Pattern	Test Real/Sim
[40]	Quadruped	X	X	2-2/3-1	Real
[45]	Hexapod	X	X	alternate tripod	Sim
[47]	Quadruped	X	X	2-2	Study
[15]	Spider	✓	X	alternate pairs	Real
[46]	Spider	✓	X	tripod	Real
[12]	Hexapod	X	X	tripod	Real
[48]	-	X	X	pattern extraction	Real
[49]	wheel-legged	X	✓	hybrid	Real
[50]	Quadruped	X	X	2-2	Sim
[43]	Quadruped	X	✓	2-2	Real
Authors	Quadruped	✓	✓	2-2/3-1	Real/Sim

**Table 7:** Robot joint nomenclature.

Joint Nomenclature					
**1. F**	Front	**2. R**	Right	**3. H**	Hip
	
				**3. T**	Thigh
**1. R**	Rear	**2. L**	Left		
				**3. C**	Calf

## Data Availability

The dataset for neural network training can be found in Appendix A.

## Abbreviations

The following abbreviations are used in this manuscript:

ARTU-R	A1 Rescue Task UPM—Robot
ROS	Robot Operating System
CNN	Convolutional Neural Netwrok
YOLO	You Only Look Once

## Appendix A

The dataset used in Neural Network Training can be found through the following link: https://github.com/ChristyanCruz11/Terrains (accessed on 1 June 2023).
